# Increasing Incidence of *Clostridium difficile*-associated Disease, Singapore

**DOI:** 10.3201/eid1409.070043

**Published:** 2008-09

**Authors:** Poh Lian Lim, Timothy M.S. Barkham, Li Min Ling, Frederico Dimatatac, Tamuno Alfred, Brenda Ang

**Affiliations:** Tan Tock Seng Hospital, Singapore (P.L. Lim, T.M.S. Barkham, L.M. Ling, F. Dimatatac, B. Ang); Clinical Trials & Epidemiology Research Unit, Singapore (T. Alfred)

**Keywords:** Clostridium difficile, pseudomembranous colitis, Asia, Singapore, incidence, epidemiology, risk factors, quinolone, letter

**To the Editor**: *Clostridium difficile*–associated disease (CDAD) has increased in incidence across North America and Europe (*1*). Recent reports document the emergence of an epidemic strain of *C. difficile*, NAP1/BI/027, associated with increased virulence (*2*,*3*). However, less information is available regarding CDAD epidemiology in Asia. We examined the incidence of *C. difficile* among hospitalized patients in Singapore from 2001 through 2006 and conducted a case–control study to evaluate risk factors for testing positive for *C. difficile* toxin (CDT) in our population.

Tan Tock Seng Hospital (TTSH) is a 1,200-bed, acute-care general hospital in Singapore that serves an urban population of 4 million. We calculated CDAD incidence using the number of patients testing positive for CDT per 10,000 patient days from 2001 through 2006. We used this calculation because CDT testing would have been ordered for clinical indications. CDT testing was performed by using the same ELISA (Premier Toxins A&B; Meridian Bioscience, Inc., Cincinnati, OH, USA) throughout the entire period of investigation.

Case-patients and controls were selected from patients hospitalized at TTSH from January 1 through December 31, 2004. Microbiology laboratory records were used to define 3 groups. Case-patients were defined as CDT-positive inpatients (group 1). Two sets of negative controls were defined: the first (group 2) consisted of patients who tested negative for CDT. However, because false-negatives could nullify differences between groups 1 and 2, we defined a second set of negative controls (group 3) from among 18,000 inpatients not tested for CDT. Seventy patients were selected from each group by using a random number generator program. Forty-eight, 61, and 56 records were retrieved for groups 1, 2, and 3, respectively. Standardized forms were used to extract data from hospital medical records. Demographic data and hospitalization details, including ward type (6-bed, 4-bed, or single room), were collected. We examined antimicrobial drug use within 30 days of admission and within 30 days of CDT testing. We also evaluated the use of proton pump inhibitors (PPIs) and H2 blockers because these have been reported as risk factors (*1*,*4*–*6*). Outcomes ascertained included the time to discharge after CDT testing, and death within 30 days after CDT testing. The study was approved by the institutional ethics review board.

Characteristics of case-patients and controls were compared by using the Wilcoxon rank sum test for continuous variables and the Fisher exact test for categorical variables. Variables significantly associated with CDT in the univariate analysis were selected for inclusion in the multivariate regression model. A 2-sided p value <0.05 was considered significant for all comparisons.

CDAD incidence rose sharply from 1.49 cases per 10,000 patient-days in 2001 to 6.64 cases per 10,000 patient-days in 2006 (Figure). During the same period, the percentage of CDT-positive samples increased from 7% to 11%, while the number of samples tested increased from 906 to 3,508.

**Figure:**
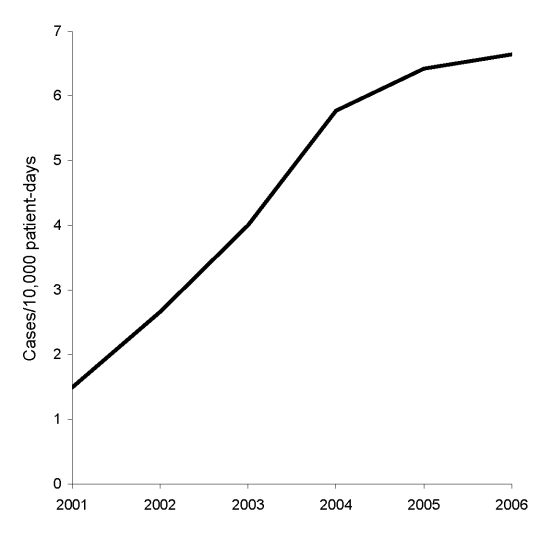
*Clostridium difficile*–associated disease incidence, Singapore, 2001–2006.

Comparing group 1 (CDT positive) with group 2 (CDT negative), a CDT-positive result was more likely to occur in those with prolonged hospital admissions (>14 days) than in those who had shorter hospital stays (<7 days; odds ratio [OR] 2.59, 95% confidence interval [CI] 1.01–6.63). Of the 19 CDT-positive patients on PPIs and the 34 CDT-negative patients on PPIs, the median exposures were 14 and 7 days, respectively (p = 0.01). In multivariate analysis, exposure to broad-spectrum antimicrobial drugs was a borderline significant risk factor (adjusted OR 2.24, 95% CI 1.00–5.02, p = 0.05).

When group 1 (CDT positive) was compared with group 3 (not tested for CDT), quinolones (OR 6.67, 95% CI 1.85–24.03), anti-anaerobic antimicrobial agents (OR 7.29, 95% CI 2.39–22.26), and stay in a 6-bed ward (OR 3.15, 95% CI 1.01–9.82) were significant risk factors in multivariate analysis. Case-patients were more likely than controls to have a longer hospital stay after testing positive. The median hospital stay after CDT testing was 16 days for case-patients versus 11 days for controls (p = 0.03).

This study documents a 4-fold rise in CDAD incidence among hospitalized patients in Singapore from 2001 through 2006. The current incidence, 6.64 per 10,000 patient-days, is comparable to that reported by large hospitals in Canada (*7*), which indicates that CDAD has emerged as an important nosocomial infection in Singapore. This incidence rate, based on the number of patients (rather than the number of isolates) who had positive CDT test results, and the rise in sample positivity from 7% to 11% suggests that the higher rates are due to a true increased occurrence rather than merely more testing.

Possible factors driving the rise in CDAD include increased use of antimicrobial agents or changes in use patterns. The volume of quinolones and broad-spectrum antimicrobial drugs used at TTSH doubled between 2002 and 2005, consistent with other studies implicating quinolones as a risk factor in CDAD (*4*).

Rising incidence or virulence could herald the geographic spread of new *C. difficile* strains. Given the spread of NAP1/BI/027 strains in other parts of the world, this increased incidence in Singapore should heighten vigilance for the introduction of outbreak strains into Asia.

The findings from this study have implications for hospital management and infection control. Environmental contamination has been described as a mode of transmission (*1*). Potential crowding in 6-bed wards may increase spread of CDAD and may be particularly relevant in busy healthcare facilities in Asia. CDAD is estimated to cost the healthcare system in the United States $3.2 billion annually (*8*). With longer hospitalization for persons after they test positive for CDT, as seen in our study, rising CDAD rates could increase hospital occupancy and result in excess healthcare expenditures.

CDAD in Asia is an emerging challenge that needs to be recognized. Its control will ultimately depend on priority being given to epidemiologic surveillance, infection control, and stewardship of antimicrobial agents.
